# Diagnosis and treatment of macrocytic anemia in a perinatal common marmoset: a case report

**DOI:** 10.1186/s42826-022-00115-6

**Published:** 2022-02-28

**Authors:** Jong-Min Kim, Jina Kwak, Hyun-Jin Lim, Joo-Il Kim, Byeong-Cheol Kang

**Affiliations:** 1 Xenotransplantation Research Center, Seoul National University College of Medicine, Seoul, Korea; 2 Institute of Endemic Diseases, Seoul National University College of Medicine, Seoul, Korea; 3 Cancer Research Institute, Seoul National University College of Medicine, Seoul, Korea; 4 Department of Experimental Animal Research, Biomedical Research Institute, Seoul National University Hospital, Seoul, 110-799 Korea; 5 Graduate School of Translational Medicine, Seoul National University College of Medicine, 101 Daehakro, Jongno-gu, Seoul, 03080 Korea; 6 Biomedical Center for Animal Resource and Development, Seoul National University College of Medicine, Seoul, Korea; 7 Designed Animal and Transplantation Research Institute, Institute of Green Bio Science Technology, Seoul National University, Pyeongchang-gun, Gangwon-do Korea

**Keywords:** Macrocytic anemia, Cobalamin, Folate, Perinatal common marmoset

## Abstract

**Background:**

The common marmoset is widely used in current biomedical research for various research fields. We observed macrocytic anemia in a perinatal common marmoset with gradual weight loss and diarrhea. The objective of this case report is to describe the diagnosis and treatment of macrocytic anemia in a perinatal common marmoset.

**Case presentation:**

A 7-year-old female common marmoset showed clinical signs of gradual weight loss and intermittent diarrhea beginning 3 months after giving birth. Macrocytic anemia was diagnosed due to a decreased red blood cell (RBC) count, low hemoglobin level, and increased mean corpuscular volume (MCV). Multivitamins containing cobalamin and folate were administered for 7 days, and the patient’s RBC count recovered to near the normal range with this treatment.

**Conclusions:**

Macrocytic anemia can be diagnosed by evaluating the MCV on a complete blood count (CBC) and cobalamin or folate levels and be treated by supplementation with cobalamin and folate. Such supplements may be needed during pregnancy and lactation in female common marmosets and/or in animals with chronic diarrhea.

## Background

Common marmosets (*Callithrix jacchus*) are New World nonhuman primates (NHPs) that have been widely used for biomedical research in neuroscience, aging, reproductive biology, behavior, and drug development and safety because of they are small in size, cost relatively little to keep, require little effort for husbandry, are easy to handle for routine clinical procedures, have rapid reproductive turnover, and have a lower incidence of zoonoses than other NHPs such as macaques or baboons [1]. Thus, veterinary management of common marmosets is important for herd control and to obtain qualifying research results.

We observed macrocytic anemia in a perinatal common marmoset with symptoms of gradual weight loss and diarrhea. Macrocytic anemia is defined an increased mean corpuscular volume (MCV) of red blood cells (RBCs) and is induced by cobalamin (vitamin B12) or folate (vitamin B9) deficiency. The objective of this case report is to report the diagnosis and treatment of macrocytic anemia in a perinatal common marmoset.

## Case presentation

The animal experiments were approved by the Institutional Animal Care and Use Committee (IACUC) of the Biomedical Research Institute at the Seoul National University Hospital (an Association for Assessment and Accreditation of Laboratory Animal Care (AAALAC)-accredited facility; IACUC number: 20-0161-S1A1). We obtained a group of common marmosets from CLEA (Tokyo, Japan). A 7-year-old female common marmoset (ID: 17Cj03) showed clinical signs of gradual weight loss (from 306 to 250 g), intermittent diarrhea, depression, and a poor hair coat beginning 3 months after giving birth. A complete blood count (CBC) and biochemical analysis were performed for diagnosis (Tables 1 and 2). Macrocytic anemia was diagnosed due to a decreased RBC count, low hemoglobin level, and increased MCV. Multivitamins (1/10 tab, P.O., SID; Hi Well Premium Multi Vitamins & Mineral for Women^®^, Hi Well Healthcare, Auckland, New Zealand, Table 3) were administered for 7 days, and a repeated CBC was performed to monitor the patient’s response to multivitamin treatment. Her RBC count recovered to near the normal range (Table 1).

**Table 1:** CBC results of 17Cj03

Parameter	Day of diagnosis of macrocytic anemia	1 week after multivitamin treatment
WBC (10^3^/μl)	5.01	6.03
Neutrophil (10^3^/μl)	1.02	2.58
Lymphocyte (10^3^/μl)	3.45	2.81
Monocyte (10^3^/μl)	0.21	0.43
Eosinophil (10^3^/μl)	0.01	0.02
Basophil (10^3^/μl)	0.27	0.02
RBC (10^6^/μl)	3.73	5.39
Hb (g/dl)	9.7	13.6
Hct (%)	36.9	47.0
MCV (fL)	98.7	87.3
MCH (pg)	25.9	25.2
MCHC (g/dL)	26.2	28.9
PLT (10^3^/μl)	765	976
Reticulocyte (%)	24.9	15.1

**Table 2:** Blood chemistry results on the day of diagnosis of macrocytic anemia in 17Cj03

Parameter	Results
TP (g/dl)	5.9
ALB (g/dl)	3.4
ALP (U/l)	1526
AST (U/l)	88
ALT (U/l)	2
BUN (mg/dl)	42.4
CRE (mg/dl)	0.49
GLU (mg/dl)	168
TBIL (mg/dl)	0.2
GGT (U/l)	4
TG (mg/dl)	72
TCHO (mg/dl)	119
LDH (U/l)	220
Na (mEq/l)	149
K (mEq/l)	4.8
Cl (mEq/l)	107
Ca (mg/dl)	7.3
P (mg/dl)	3.8

**Table 3:** Ingredients of 1 tab of multivitamins

Ingredients	Content	Ingredients	Content	Ingredients	Content
Vitamin A	530 μg RE	Vitamin C	350 mg	Magnesium	25 mg
Vitamin B1	19 mg	Vitamin D3	9 μg	Manganese	1 mg
Vitamin B2	2.8 mg	Vitamin E	21 mg	Selenium	47 μg
Vitamin B3	15 mg NE	Biotin	39 μg	Chromium	135 μg
Vitamin B5	22 mg	Rutin Trihydrate	25 mg	Copper	0.5 mg
Vitamin B6	7.5 mg	Calcium	10 mg	Zinc	12 mg
Vitamin B12	6 μg	Iron	12 mg	Encapsulating aids	
Folic acid	600 μg	Iodine	160 μg		

## Discussion and conclusions

Macrocytic anemia is divided into two forms, megaloblastic (hypersegmented neutrophils) and non-megaloblastic. The megaloblastic form is due to impaired DNA synthesis from folate and/or vitamin B12 deficiencies, while the non-megaloblastic moiety (absence of hypersegmented neutrophils) occurs from multiple mechanisms such as alcohol consumption (RBC toxicity), hereditary spherocytosis (impaired volume regulation increases red cell size), hypothyroidism and liver disease (due to lipid deposition in the cell membrane), and marked reticulocytosis from states of excess RBC consumption such as hemolysis or turnover in pregnancy or primary bone marrow disease [2]. A limitation of this case report is that we could not identify hypersemented neutrophils by blood smear at the time of anemia, so we could not differentiate macrocytic anemia into megaloblastic and non-megaloblastic forms in this case. If the CBC test shows macrocytic anemia, it will be very important to differentiate it by blood smear. However, since it was confirmed that the anemia was improved by the administration of vitamin B12 and folate, it can be said that this case was in megaloblastic form of macrocytic anemia.

The megaloblastic form of macrocytic anemia is induced by a low serum cobalamin or folate level. Evaluation of serum cobalamin and folate levels requires 0.8 ml of serum (1.6 ml of whole blood) in our test protocol. This is a relatively large blood volume to collect in a common marmoset, especially in an anemic animal. Thus, to evaluate serum cobalamin and folate levels, we alternatively selected an archived sample that was collected from a euthanatized 7-year-old female common marmoset (ID: 15Cj08) with clinical signs of gradual weight loss (from 269 to 224 g), chronic diarrhea, anemia, and the same history of disease onset after childbirth. This marmoset’s cobalamin and folate levels were 133 pg/ml and > 120 ng/ml, respectively (Centaur XP, Siemens, USA). The reference ranges for these parameters from Parambeth et al.’s group were 322–2642 pg/ml for cobalamin and 54.8–786.4 ng/ml for folate [3]. Although this marmoset (15Cj08) did not show macrocytic anemia, cobalamin deficiency was detected. The reason why this marmoset (15Cj08) did not show macrocytic anemia despite having a low cobalamin level remains unknown. One of the possibilities may be that when less than 100 pg/ml of cobalamin level in humans is diagnosed as macrocytic anemia by cobalamin deficiency, so this individual is judged to be at the borderline of cobalamin deficiency. However, the patient in this case (17Cj03) likely had macrocytic anemia induced by cobalamin deficiency because she was in the same herd and had the history of disease onset after childbirth, clinical signs, and diet. In women, cobalamin deficiency during pregnancy and lactation is a public health problem in populations with low consumption of animal products, and balanced intake of cobalamin-folate is necessary during pregnancy and lactation to prevent unhealthy consequences [4]. Since the common marmosets in this report were also pregnant and lactating, they may have needed cobalamin and folate supplementation similar to humans.

Another reason for low cobalamin and folate levels is chronic diarrhea. Intestinal absorption of cobalamin occurs via several processes, and absorption of a complex of cobalamin and intrinsic factors occurs through specific receptors in the enterocytes of the ileum [5]. Ileal disease leads to damage or decreased expression of cobalamin receptors, ultimately leading to decreased absorption of cobalamin and depletion of the body's reserves. In addition, intestinal microbial imbalances caused by disease can reduce serum cobalamin levels because many bacteria compete to bind intestinal available cobalamin [6]. Folate is another water-soluble vitamin of the B-complex family. Chronic bowel disease impairs folate carriers, which reduces folate intake, leading to decreased serum folate concentrations [7]. Diarrhea was also present in this case, and it is thought that this diarrhea may have contributed to the deficiency of cobalamin and folate. Another differential diagnosis based on this marmoset clinical symptom (intermittent diarrhea and weight loss) is wasting marmoset syndrome (WMS). WMS is a disease unique to this species and its main symptoms include weight loss, decreased muscle mass, and chronic diarrhea [8]. In marmosets with symptoms of WMS, megaloblastic form of macrocytic anemia may occur, so careful management is necessary.

In conclusion, macrocytic anemia can be diagnosed by evaluating the MCV on a CBC and cobalamin or folate levels and be treated by supplementation with cobalamin and folate. Such supplements may be needed during pregnancy and lactation in female common marmosets and/or in animals with chronic diarrhea.

## Data Availability

Not applicable.

## Abbreviations

AAALAC: Association for Assessment and Accreditation of Laboratory Animal Care
CBC: Complete blood count
IACUC: Institutional Animal Care and Use Committee
MCV: Mean corpuscular volume
WBC: White blood cell
